# Perioperative Complications in Percutaneous Nephrolithotomy: Predictive Risk Factors and Hemodynamic Alterations

**DOI:** 10.7759/cureus.69488

**Published:** 2024-09-15

**Authors:** Milind Kothiyal, Nidhi Kumar, Gurjeet Khurana

**Affiliations:** 1 Department of Critical Care, Graphic Era Medical College, Dehradun, IND; 2 Department of Anesthesia, Himalayan Institute of Medical Science, Swami Rama Himalayan University, Dehradun, IND

**Keywords:** blood transfusion, fine needle aspiration, fluctuating blood pressure, nephrolithotomy, temperature alteration

## Abstract

Background

Percutaneous nephrolithotomy (PCNL) is one of the most frequently used methods of treating large and/or complex kidney stones. Despite the growing interest in this area, there are relatively few papers that report studies of the changes in hemodynamics in the perioperative period and the potential factors that might influence the patient’s condition. This aspect, however, has not been well described in the current literature, although fluctuations in blood pressure, temperature, and electrolyte balance during surgery may also lead to development of complications.

Objectives

In this study, variations in vital signs during surgery and between surgeries of 134 patients who underwent PCNL were analyzed in relation to other predisposing factors such as hypothermia and the need for blood transfusion.

Methods

Biochemical data such as systolic blood pressure (SBP) and diastolic blood pressure (DBP), temperature, hemoglobin (Hb), hematocrit (Hct), and serum sodium and potassium were assessed at admission, during PCNL, and at 24 hours after surgery in 134 patients. In this study logistic regression was used to assess the influence of factors such as age, BMI (body mass index), surgery duration, volume of irrigation fluid, number of tracts, estimated blood loss, hypothermia, and requirement of red blood cell transfusions.

Results

The mean arterial pressure declined during surgery as compared to the preoperative value, reaching a value of 97.34 during the operation. The monitored mean temperature of the patient during the perioperative period was therefore reduced, with a small increase towards the end of the surgery. In the plasma concentrations, there was a decrease compared to pre- and postoperative values, and there was also a decline in 48-hour Hb, Hct, and sodium values. The analysis identified the volume of irrigation fluid as a predictor of mild hypothermia risk. A longer surgery time along with more tracts and worse condition of the patient meant that the patient needed more blood transfusion. It was also noted that none of them influenced the amount of Hb that was lost.

Conclusion

Variability in the recorded ABCs (airway, breathing, and circulation) before and after PCNL procedures was normative. Despite hypothermia being associated with the volume of irrigation fluids used in surgeries, most of the transfusions were administered in patient-complicated operations and critical states. This suggested that there was potential in defining the modifiable factors that worsen clinically reported outcomes to assist in enhancing protocol.

## Introduction

Kidney stones are a common problem, affecting 5-12% of the global population [1]. The various factors responsible for renal stones include environmental, nutritional, and metabolic changes, and these are the major reasons for the rising trend in the developed world [2]. percutaneous nephrolithotomy (PCNL) is a surgery that offers advantages such as decreased hospital stay, less pain, early ambulation, and reduced injury to the kidney [3,4]. With the recent technical advancements, most calculi are now treated percutaneously using minimally invasive endoscopic techniques [5]. It is the standard of care for staghorn calculi, large calculi (>2 cm), upper urinary tract calculi, difficult lower pole stones, cystine stones, and stones in abnormal kidneys [6].

PCNL is less invasive (>90% success rate), but the complication rate is still >10% [7]. There are both predictable and unpredictable complications, including hemorrhage, hypothermia, fluid overload, collecting system injuries, sepsis, stricture formation, damage to nearby organs, technical complications during surgery, neurocutaneous fistula, renal loss, and even death [8]. True complication rates of PCNL are challenging to determine accurately because most studies focus only on procedure-specific outcomes [9-12]. Three major complications associated with PCNL are bleeding, hypothermia, and cardiovascular changes. Body heat loss increases during surgery due to altered thermoregulation, influenced by anesthetic drugs, presurgical preparations, and patient positioning [13].

Studies show significant temperature drops during PCNL surgery. Continuous irrigation at room temperature during surgery can lead to hemodilution, fluid overload, and heat loss due to intravascular absorption via injured veins and spillage into the perinephric space [14,15]. High-risk surgical patients are particularly susceptible to inadvertent core hypothermia in the immediate postoperative period. In addition to this, anesthesia also impairs the central thermoregulation system, allowing redistribution of body heat [16]. Hypothermia may then cause changes in coagulation times [17]. Spinal anesthesia (SA) has many advantages over general anesthesia (GA) for PCNL surgeries. It may lower postoperative pain and reduce analgesic drug consumption, thereby avoiding other side effects from GA medications [18]. Therefore, we conducted this study to assess the outcome of PCNL under SA by analyzing its risk factors for perioperative complications.

## Materials and methods

Study design and ethics

The study was conducted as a prospective, cohort study to evaluate the results of the intervention in the subsequent year. The study received approval from the Institutional Ethics Committee of the Himalayan Institute of Medical Sciences, Dehradun (SRHU/HIMS/RC/2020/03). Patients were enrolled using a systematic, non-sequential method to minimize selection bias. Recruitment entailed identifying patients who met the inclusion criteria for informed consent, which was quite difficult, especially during the early stages. The introduction of a small monetary incentive, as an amendment, subsequently increased enrollment rates. Overall, an observational study design was followed, and the procedural measures were effective when evaluating the key outcome parameters in the 12 months of follow-up.

Participants

A cross-sectional study was conducted on 134 patients from the hospital who were 15-59 years old and underwent unilateral PCNL. Eligible patients had a hemoglobin (Hb) level greater than 10 g/dL and were classified as American Society of Anesthesiologists (ASA) physical status class I or II. The following factors were considered as exclusion criteria for patients: anemia, chronic kidney disease, dialysis dependence, history of cardiac disease, bilateral percutaneous nephrostomy tubes, recent transfusion, prior percutaneous nephrostomy/surgery, or current use of tranexamic acid. The inclusion and exclusion criteria were designed to provide a methodologically sound patient sample, free from significant comorbidities that could impact the success of the unilateral PCNL procedure.

Preoperative assessments

The patients were checked for overall health, and some tests were conducted on the patients as part of a check-up. Data on age, treatment history, presenting complaints, clinical signs, baseline Hb, packed cell volume, and serum electrolytes were accurately recorded. In addition to the type of stone, demographic data including age, sex, and body mass index (BMI) were collected, as well as stone characteristics including right or left and size. In general, all the required investigations were performed, basic physiological investigations were conducted, and important information pertinent to the stone was recorded in anticipation of its further handling and management.

Anesthesia and monitoring

The respondents were premedicated for surgery and fasted in a way that was appropriate for age. A peripheral line was inserted, and Ringer’s lactate was commenced for resuscitation purposes. Additional parameters monitored continuously included ECG, non-invasive blood pressure, and pulse oximetry. Rectal thermometry was used to measure the patient’s core temperature and to identify any changes that may indicate fever. For surgery, the anesthesiologist was able to use a spinal anesthetic, SA2B subarachnoid block with heavy bupivacaine. This approach offered sufficient sensory anesthesia for the abdominal surgery while the patient can breathe spontaneously. Supervision of all variables was maintained throughout the process to assess the quality and safety of the anesthesia administered. Surgery and anesthesia were uncomplicated, and the patients were transferred to the recovery room, following the surgery in good condition.

Intraoperative details

The position of the patient for the procedure was either supine or as required based on the access needed. Special emphasis was placed on regulating the body's temperature throughout the procedure. The temperature of the irrigating fluids and warming equipment were kept close to normal body temperature (Tfluid = Tbody). Temperature recording intervals were set every 15 minutes (Δt = 15 minutes), and these measurements were carefully documented. Additionally, the procedure time was recorded along with the amount of iced lavage applied. Specific observations regarding the surgical tract, including its depth, width, and length, were meticulously recorded. Other features of the tract, such as the type of tissue encountered, were also noted. Rigorous documentation of all procedures was crucial for assessing patient stability and surgical outcomes. Accurate notes allowed the surgical team to verify that their treatment adhered to the established standards and to identify any areas needing improvement.

Postoperative assessments

The patient’s hemodynamic and temperature values were also assessed in the post-anesthesia care unit (PACU) after the surgery. After the surgery, the patient’s temperature was checked and maintained to the normal range. As for the patient, the postoperative serum Hb, hematocrit (Hct), and electrolyte levels were evaluated for the possibility of bleeding or electrolyte disorders. In the first week after the surgery, the management primarily consisted of evaluating and treating any hemorrhage or other post-surgical issues. The patient’s condition was closely observed in the PACU to determine their status back to baseline after the anesthetic and surgical stressors.

Statistical analysis

The data were collected and entered into Microsoft Excel 2010 (Microsoft Corp., Redmond, WA). Statistical analysis was performed using SPSS Version 22 (IBM Corp., Armonk, NY). Paired t-test was used to compare pre- and postoperative Hb, Hct, and electrolyte levels. The Wilcoxon signed-rank test was used to compare hemodynamic and thermal parameters. Univariate logistic regression analysis was used to evaluate the potential relationships between bleeding, hypothermia, and transfusion needs, along with clinical and other procedural characteristics. The level of significance, usually denoted as α\alphaα, had the following criterion: if p<0.05p, then the hypothesis was considered significant.

The sample size for the study was calculated using the formula:

$n = \frac{Z_{\alpha/2}^2 \cdot p \cdot q}{l^2}$

where 

$Z_{\alpha/2}$= 1.96

p = 56.2 (analysis and assessment of factors affecting bleeding in percutaneous nephrolithotomy under SA) (3).

q = 43.8

l = 15% relative error (15% of P)

The calculated sample size was n = 134.

## Results

Demographics and clinical characteristics

**Figure 1:**
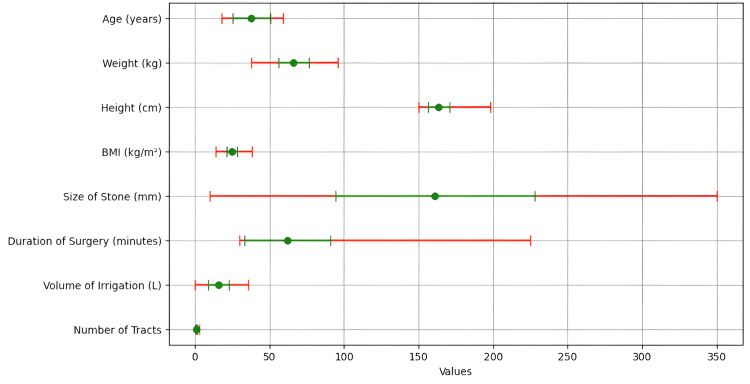
Comparative analysis of clinical and procedural variables

The study targeted 134 patients, comprising 26.87% female patients ( n=36) and 73.13% male patients (n=98). The mean age of the participants was 45 years, with an age range of 18 to 59 years. The mean body weight was 72 kg, with a minimum of 38 kg and a maximum of 96 kg. The mean height was 170 cm, and the mean BMI was 24.5. The size of the stone, measured in millimeters, had a mean value of 66.7 mm (range: 10 to 350 mm). The overall average duration of surgery was 120 minutes (range: 30 to 225 minutes), and the mean irrigation volume was 2.5 liters (range: 0.25 to 36 liters). The patients underwent surgery through an average of 1.14 tracts per patient (minimum: 1, maximum: 3 tracts) with tract sizes ranging from 24F to 30F, as given in Table 1. A graph with mean values and confidence intervals for various clinical and procedural variables, such as age, weight, height, BMI, stone size, duration of surgery, irrigation volume, and the number of tracts, is depicted in Figure 1.

**Table 1:** Demographic data Gender: male (n=98, 73.13%) and female (n=36, 26.87%) Tract size: the tract size was documented for each procedure, providing key insights for the urology community regarding its influence on patient outcomes. BMI, body mass index

Variable	Mean ± SD	Range
Age (years)	37.98 ± 12.57	18-59
Weight (kg)	66.28 ± 10.15	38-96
Height (cm)	163.61 ± 7.29	150-198
BMI (kg/m²)	24.76 ± 3.45	14.17-38.46
Size of stone (mm)	161.1 ± 66.7	10-350
Duration of surgery (minutes)	62.26 ± 28.9	30-225
Volume of irrigation (L)	16.2 ± 6.95	0.25-36
Number of tracts	1.14 ± 0.44	1-3

Hypotensive and hypertensive episodes during surgery

The changes in systolic blood pressure (SBP) and diastolic blood pressure (DBP) at various time points during the surgical procedure of 134 patients are presented in Table 2. Compared with the baseline value, SBP was reduced after SA and at most of the time points up to 2 hours postoperatively (p = 0.0001 to p = 0.041). The only three values measured starting from 2 hours and 15 minutes were not significantly different from the baseline values. For DBP, there were significant changes compared to baseline at all time points up to 1:45 hours (p < 0.0001 to p = 0.045). Differences in DBP from baseline and later time points were not significant at 2 hours or beyond. Based on the results, it can be concluded that SA and surgery caused a significant reduction in both SBP and DBP, which was maintained for most of the first hour during the surgery but also trended back towards baseline values a few hours later. The drops were more prominent and lasted for a longer time for SBP compared to DBP.

**Table 2:** Comparison of SBP and DBP at different time intervals in mm Hg (n=134) Significance value: p<0.05 DBP, diastolic blood pressure; SBP, systolic blood pressure

	SBP		DBP	
Time interval	Mean ± SD	P-value	Mean ± SD	P-value
Baseline	123.28 ± 14.98	-	75.48 ± 8.98	-
Post-spinal SBP	109.68 ± 12.52	<0.0001	68.81 ± 7.75	<0.0001
15 minutes	109.46 ± 11.93	<0.0001	70.1 ± 7.27	<0.0001
30 minutes	105.74 ± 9.66	<0.0001	69.52 ± 6.47	<0.0001
Prone SBP	101.54 ± 10.16	<0.0001	66.56 ± 7.73	-
45 minutes	108.75 ± 0.22	<0.0001	69.9 ± 6.81	<0.0001
1 hour	110.06 ± 11.93	<0.0001	70.51 ± 7.67	0.0002
1:15 hours	112.38 ± 9.8	<0.0001	72.16 ± 7.33	0.002
1:30 hours	111.12 ± 2.88	0.002	72.06 ± 8.41	0.011
1:45 hours	113.08 ± 11.35	0.010	72.39 ± 7.79	0.045
2 hours	115.36 ± 12.26	0.041	72.09 ± 6.11	0.123
2:15 hours	112.17 ± 9.97	0.078	70.33 ± 5.92	0.173
2:30 hours	114.2 ± 11.58	0.461	70.6 ± 3.97	0.345
End of procedure	112.54 ± 10.02	0.224	71.43 ± 6.91	-

Temperature changes during surgery

In this study, temperature was measured in 134 patients undergoing surgery. The mean temperature at baseline measurement was 37.34°C, which became 37.44°C after SA with a non-significant change from the baseline (p = 0.245). It subsequently decreased at 15 minutes (37.11°C, p < 0.0001), at 30 minutes (36.58°C, p < 0.0001), during prone positioning (36.71°C, p < 0.001), at 45 minutes (36.13°C, p < 0.0001), at 1 hour (35.97°C, p < 0.0001), and at all subsequent measurements compared to the baseline. The lowest mean temperature was recorded at the time interval of 1 hour 30 minutes (35.62°C). We observed a statistically significant rise in temperature compared to baseline at 2 hours 15 minutes (35.90°C, p = 0.028) and 2 hours 30 minutes (36.00°C, p = 0.042), though it was still subnormal. The mean temperature at the end of the procedure was 35.94°C, as given in Table 3. In general, temperature was lower by the end of the surgery than at baseline and increased slightly at the end of the surgery.

**Table 3:** Comparison of temperature at different time intervals (n=134) Significance value: p<0.05

Variables	Temperature	
	Mean ±SD	P-value
Baseline	37.34 ± 0.34	-
Post-spinal SBP	37.44 ± 0.39	0.245
15 minutes	37.11 ± 0.4	<0.0001
30 minutes	36.58 ± 0.59	<0.0001
Prone SBP	36.71 ± 0.51	<0.001
45 minutes	36.13 ± 0.73	<0.0001
1 hour	35.97 ± 0.64	<0.0001
1:15 hours	35.74 ± 0.65	<0.0001
1:30 hours	35.62 ± 0.6	0.000
1:45 hours	35.72 ± 0.7	0.001
2 hours	35.81 ± 0.78	0.005
2:15 hours	35.90 ± 0.98	0.028
2:30 hours	36.00 ± 1.08	0.042
End of procedure	35.94 ± 0.73	-

Difference in the blood level pre- and postoperatively

Based on the author's comment, the revised text should emphasize the risk factor responsible for the changes in the blood parameters rather than attributing them directly to surgery. A comparison test was conducted on 134 patients to evaluate how a specific risk factor affected their Hb, Hct, sodium, and potassium levels before and after surgery, as shown in Table 4. A significant difference was observed in Hb and Hct levels (p<0.001) due to this risk factor. The mean Hb value decreased from 13.44 g/dL to 12.32 g/dL, representing a difference of 1.12 g/dL (an 8.33% decrease). Similarly, Hct dropped from 40.23% to 36.91%, reflecting a difference of 3.32% (an 8.25% decrease). Sodium levels also showed a marginal reduction from 136.75 mEq/L preoperatively to 135.56 mEq/L postoperatively (p = 0.05), with a difference of 1.19 mEq/L (a 0.87% decrease). Potassium levels, however, remained stable, with a mean of 4.17 ± 0.59 mEq/L before surgery and 4.08 ± 0.47 mEq/L afterwards (p = 0.34), representing a difference of 0.09 mEq/L (a 2.16% decrease that is not statistically significant). These findings indicate that the risk factor under study, rather than the surgery itself, primarily contributed to the changes in Hb, Hct, and sodium levels within 24 hours, while potassium levels were unaffected.

**Table 4:** Pre- and postoperative Hb, HCT, sodium, and potassium levels (n=134) Significance value: p<0.05 *Paired t-test Hb, hemoglobin; HCT, hematocrit

Variables	Hb	HCT	Sodium level	Potassium level
	Mean ± SD	Mean ± SD	Mean ± SD	Mean ± SD
Before 24 hours	13.44 ± 1.91	40.23 ± 6.4	136.75 ± 3.37	4.17 ± 0.37
After 24 hours	12.32 ± 2.14	36.91 ± 6.74	135.56 ± 3.18	4.08 ± 0.47
p-value	<0.001*	<0.001*	<0.05*	0.34

Hypothermic patients

To investigate the variables that are significantly related to the development of hypothermia, univariate logistic regression analysis was run with 134 patients in the hypothermia group. The following factors were used as predictors: age of the patient, duration of surgery, the volume of irrigation used, request for blood transfusion, number of tracts created, BMI, and amount of blood lost. Despite the study, it was only found that the volume of irrigation was found to correlate with hypothermia (p<0.0001). It was observed that there was a significant reduction in hypothermia among patients who received more than 20L of irrigation, with the odds ratio being only 0.066 (95% CI 0.025-0.177). Thus, the presence of other variables such as age, duration of surgery, blood transfusion request, number of tracts, BMI, and blood loss was not significantly related to hypothermia risk (p>0.05). In terms of the blood transfusion request, an odds ratio of 10.44 was estimated, whose confidence interval was very large (0.069-189.308) and the finding was non-significant (p = 0.256). While irrigation volumes above 20L had lower odds of perioperative hypothermia, the model found that other factors in the dataset were most predictive of temperature change, as given in Table 5. The study may have been insufficiently powered to reveal other clinical parameters’ impacts on body temperature regulation.

**Table 5:** Analysis of hypothermia with other parameters (n=134) Significance value: p<0.05 B, coefficient; S.E., standard error; CI, confidence interval

Variables		B	S.E.	P-value	Odds ratio	95% CI for odds ratio	
						Lower	Upper
Age	≤40	-	-	-	-	-	-
	>40	0.399	1.425	0.779	1.491	0.091	24.355
Duration of surgery (in minutes)	0-60	-	-	-	-	-	-
	61-120	1.067	1.184	0.373	2.908	0.230	36.763
	>120	1.578	1.788	0.412	4.846	0.032	100.987
The volume of irrigation (in liters)	≤20	-	-	-	-	-	-
	>20	-2.715	0.501	<0.0001	0.066	0.025	0.177
Request of transfusion	-	2.346	2.003	0.256	10.44	0.069	189.308
Number of tracts	Single	-	-	-	1	-	-
	Multiple	-0.864	1.846	0.64	0.421	0.011	15.718
BMI (kg/m^2^)	-	0.175	0.175	0.317	1.191	0.846	1.677
Blood loss	-	-0.956	1.658	0.586	0.385	0.028	54.534

Risk indicators involving blood transfusion

The patient data from 134 patients were analyzed using univariate logistic regression analysis to determine factors that may be linked with blood transfusion needs, as given in Table 6. Likewise, hypothermia in the prone position was no longer a risk factor for transfusion when tested independently (OR 10.44, 95% CI 0.069 to 189.308; p = 0.256). Additionally, age more than 40 years (OR 1.491, 95% CI 0.091-24.355, p = 0.779) and irrigation volume of more than 20 liters (OR 3.258, 95% CI 0.198-53.624, p = 0.409) did not translate into a higher probability of requiring transfusion support. However, surgery duration greater than 120 minutes had 10 times greater odds of having a transfusion (OR 10.200, 95% CI 0.832-92.173, p = 0.029) as compared to surgeries taking 0-60 minutes. Furthermore, the creation of additional tracts during surgery, as well as a higher ASA classification (ASA III and IV), significantly increase the estimated probability of requiring a blood transfusion. In more detail, for each additional tract, the odds ratio (OR) was 13.89 (95% CI 1.257-153.526; p = 0.032), while for patients in ASA classes III and IV, the OR was 4.297 (95% CI 1.171-15.776; p = 0.029). The other factors assessed did not correlate with transfusion needs in this patient group population.

**Table 6:** Analysis of requirement of transfusion with other parameters (n=134) Significance value: p<0.05 B, coefficient, S.E., standard error; CI, confidence interval

Variables		B	S.E.	P-value	Odds ratio	95% CI	
						Lower	Upper
Age	≤40	2.346	2.003	0.256	10.44	0.069	189.308
	>40	-	-	-	-	-	-
	-	-	-	-	1	-	-
Duration of surgery in minutes	0-60	0.399	1.425	0.779	1.491	0.091	24.355
	61-120	-	-	-	-	-	-
	>120	-	-	-	1	-	-
The volume of irrigation in liters	-	0.546	1.065	0.613	1.726	0.154	13.465
	≤20	2.322	1.185	0.029	10.200	0.832	92.173
	>20	-	-	-	-	-	-
Hypothermia (prone)	-	-	-	-	1	-	-
		1.181	1.429	0.409	3.258	0.198	53.624
		2.631	1.226	0.032	13.89	1.257	153.526

Variables linked to bleeding/hemoglobin loss

In the present study, logistic regression was applied to determine whether patient age, surgery time, and irrigation volume influenced the likelihood of bleeding/Hb loss, as shown in Table 7. Gender was divided into male and female categories, and age was categorized into less than or equal to 40 years and more than 40 years. Time taken during surgery was grouped into three categories: less than or equal to 60 minutes, 61 to 120 minutes, and more than 120 minutes. The amount of irrigation fluid used was categorized as 20 liters or less and more than 20 liters. The analysis revealed a relationship between Hb loss (bleeding) and patients age older than 40 years (OR 1.378, 95% CI 0.33-5.77, p = 0.057), as well as with a surgery duration of 61-120 minutes or longer (OR 2.105, 95% CI 0.45-20.32, p = 0.318). Further research may be required to define the impact of these parameters on increased bleeding risk during surgeries.

**Table 7:** Analysis of bleeding/hemoglobin loss with other parameters (n=134) Significance value: p<0.05 B, coefficient; SE, standard error, CI, confidence interval

Variables		B	S.E.	P-value	Odds ratio	95% CI	
						Lower	Upper
Age	≤ 40	-	-	-	1	-	-
	>40	0.321	0.73	0.66	1.378	0.33	5.766
Duration of surgery (in minutes)	0-60	-	-	-	1	-	-
	61-120	0.744	0.921	0.378	2.105	0.447	20.318
	>120	0.233	1.626	0.874	1.263	0.129	169.956
The volume of irrigation (in liters)	≤20	-	-	-	1	-	-
	>20	0.97	1.081	0.3693	2.638	0.317	21.939
	-	-0.956	1.927	0.586	0.385	0.028	54.534

## Discussion

PCNL is the current gold standard for the management of large kidney stones due to its relative safety compared to open stone surgery. SA was preferred as it ensures a shorter duration of operation, shorter hospital stay, and lower morbidity, and there is lesser need for blood transfusions and postoperative narcotic analgesics by allowing efficient management of bleeding and pain. Complication rates were similar in patients receiving GA and SA. However, the operation time was much less in the SA group [19]. In our study, hypothermia was associated with the use of more than 20 liters of irrigation fluid (p<0.0001). There was no significant association of hypothermia with age, BMI, duration of surgery, requirement of transfusion, and blood loss. The amount of irrigation fluid greater than 20 liters was a statistically significant factor related to hypothermia during surgery. A positive correlation was established between the need for blood transfusion, which was needed in four patients, and the surgery duration of more than 120 minutes. Similarly, a significant correlation between blood transfusion and the number of tracts made during surgery was seen. Operation interval and irrigation fluid seemed to cause an increased risk of bleeding intraoperatively, but when multiple logistic regression was applied, these factors showed no significance statistically.

PCNL surgery is associated with several complications. The common complications in our study were shivering in five cases, followed by postoperative fever in four patients, pneumothorax in two patients, and requirement of transfusion in two patients. There were common complications of pain in five cases, bradycardia in four cases, shivering in two cases, and fever in one case [20]. Minor complications included transient fever (32.1%), clinically insignificant bleeding (7.6%), or both (3.2%) [21]. The overall complication rate was lower in our study as compared to that of other studies.

In the present study, mean preoperative Hb and Hct were 13.44 ± 1.91 g/dL and 40.23 ± 6.4 g/dL, respectively, while the postoperative Hb and Hct were 12.32 ± 2.14 g/dL and 36.91 ± 6.74 g/dL, respectively (p<0.001). The mean drop of Hb in our study was relatively lower as compared to other studies because of the shorter duration of the procedure, smaller stone size, and the procedure being done under SA. In a study on PCNL, the mean Hb level before the operation was 13.95 ± 1.68 g/dL, which decreased to 11.94 ± 1.93 g/dL postoperatively, with the difference being statistically significant (p=0.0001) [22].

In our study, serum sodium levels (Na) showed a significant change from the preoperative period to the postoperative period. There were variations in renin, aldosterone, and adrenocorticotropic hormone levels during PCNL procedures This was in discordance with the study by Ranjan et al., who compared the preoperative, intraoperative, and postoperative sodium and potassium levels and found the difference to be insignificant [23]. The mean baseline temperature in our study was 37.34°C ± 0.34, and there was a significant fall in temperature noted from 15 minutes after SA till the end of surgery (p< 0.05). Redistribution of body heat, evaporative losses, the heat required to warm intravenous fluids, and heat loss during surgical preparations cause a drop in the body's intraoperative core temperature [24].

Mean baseline SBP in the pre-spinal period was 123.28 ± 14.98 mm Hg and was found to be lower than baseline at all time intervals until 2 hours and 30 minutes. Similarly, the mean preoperative diastolic blood pressure was 75.48 ± 8.98 mm Hg and was found to be significantly lower from post-spinal period until 1:45 hours (p<0.05). Loss of sympathetic tone and subsequent systemic vasodilation, with decreased venous return and consequent reduction in preload and afterload, resulted in a fall in blood pressure after SA, which was statistically significant in our study for most of the intraoperative time. A statistically significant change in SBP and DBP was noted in the SA group at different time intervals during the intraoperative period, which indicates the hemodynamic impact of SA on blood pressure regulation [25].

In our study, the mean stone size was 1.61 ± 0.67 cm. The average stone size in our study was smaller than that of others, and thus the average duration of surgery for the cases was relatively less. With a mean stone size of 26.64 ± 14.39 mm and a mean operation time of 95.14± 26.57 minutes [26], the mean stone size in the SA group was 31.9 ± 7.4 mm and the mean operative time was 94.00 ± 8.1 minutes [27-28]. The commonest comorbidity was hypertension (59.45%), followed by diabetes among 32.43% and hypothyroidism (5.4%), and the other rare co-morbidity was rheumatoid arthritis (2.7%). In another study, the commonest comorbidity was hypertension, which was seen in 19.5% of cases, and in our study, diabetes was seen in 11.5% of cases. Our findings reveal that renal stones can be a risk factor for hypertension but not vice versa.

Limitations of the study

The current study used a retrospective design, which is not ideal but would not be considered problematic. If a prospective design had been used, then it would have been considered better. Very few pre- and postoperative vital indicators were measured; if other vital indicators are considered, then more studies can be conducted. Further studies, including additional multivariate regression analyses with control of other potential confounding factors, would improve the understanding of risk factors for the condition. As such, more studies are required on the long-term follow-up in terms of postoperative changes. Finally, the relationships between irrigation volume and hypothermia risk were explained, yet the exact process of how they were related requires further study. Further mechanistic work is necessary to elaborate upon this observation to establish better irrigation regimens.

## Conclusions

This study highlights key risk factors for perioperative complications in patients undergoing PCNL, with a focus on bleeding, hypothermia, and hemodynamic fluctuations. The findings of the study demonstrated that SA, procedural factors such as tract size and volume of irrigation, and patient characteristics including BMI and stone size significantly impact surgical outcomes. Specifically, intraoperative hypothermia was associated with irrigation volume, while bleeding risks were tied to reductions in Hb and Hct levels. These insights can guide clinicians in optimizing patient care by identifying at-risk individuals and implementing tailored preoperative and intraoperative strategies to mitigate complications.
